# An augmented reality sign-reading assistant for users with reduced vision

**DOI:** 10.1371/journal.pone.0210630

**Published:** 2019-01-16

**Authors:** Jonathan Huang, Max Kinateder, Matt J. Dunn, Wojciech Jarosz, Xing-Dong Yang, Emily A. Cooper

**Affiliations:** 1 Department of Computer Science, Dartmouth College, Hanover, New Hampshire, United States of America; 2 Department of Psychological and Brain Sciences, Dartmouth College, Hanover, New Hampshire, United States of America; 3 School of Optometry and Vision Sciences, Cardiff University, Cardiff, United Kingdom; Purdue University, UNITED STATES

## Abstract

People typically rely heavily on visual information when finding their way to unfamiliar locations. For individuals with reduced vision, there are a variety of navigational tools available to assist with this task if needed. However, for wayfinding in unfamiliar indoor environments the applicability of existing tools is limited. One potential approach to assist with this task is to enhance visual information about the location and content of existing signage in the environment. With this aim, we developed a prototype software application, which runs on a consumer head-mounted augmented reality (AR) device, to assist visually impaired users with sign-reading. The sign-reading assistant identifies real-world text (e.g., signs and room numbers) on command, highlights the text location, converts it to high contrast AR lettering, and optionally reads the content aloud via text-to-speech. We assessed the usability of this application in a behavioral experiment. Participants with simulated visual impairment were asked to locate a particular office within a hallway, either with or without AR assistance (referred to as the AR group and control group, respectively). Subjective assessments indicated that participants in the AR group found the application helpful for this task, and an analysis of walking paths indicated that these participants took more direct routes compared to the control group. However, participants in the AR group also walked more slowly and took more time to complete the task than the control group. The results point to several specific future goals for usability and system performance in AR-based assistive tools.

## Introduction

Navigation is a multifaceted task, for which people typically rely heavily on vision [1,2]. Two basic requirements for effective navigation are wayfinding (using environmental information to move toward a destination) and obstacle avoidance. Both wayfinding and obstacle avoidance can pose challenges for individuals with vision loss [3]. At the same time, navigation and free mobility are key to carrying out the tasks of day-to-day life independently. Thus, technologies that can effectively assist people to navigate with limited vision may have a significant impact on the quality of life of millions of people [4].

While existing aids such as canes and GPS technology [5–7] can assist with obstacle avoidance and outdoor wayfinding, their applicability for indoor wayfinding is limited. To address this issue and expand on current navigational aids, researchers have proposed and developed tools that use off-the-shelf mobile devices, such as smartphones, to provide positional information and wayfinding guidance indoors (e.g., [8–13]). For example, direct sensing techniques may be used in which interactive tags are placed within an indoor environment [10,12–14]. Such techniques utilize radio frequency identification (RFID) or Bluetooth beacons to label spaces. Other proposed approaches for indoor wayfinding aids use computer vision to identify a user’s location based on their surroundings [9,15,16] or employ annotated maps combined with user input [8]. However, these techniques either require collecting and storing custom site-specific information on a server or are not currently fully implemented. A recent study reported the development of a smartphone application to assist specifically with backtracking an indoor path, which operates using inertial sensors and does not require any preexisting maps [17].

For individuals who have limited vision but are still able to read magnified text, another possibility for assisting indoor wayfinding is to provide the user with enhanced visual information about the location and content of existing signage in the environment. For this purpose, systems that utilize head-mounted displays (HMDs) may offer unique features: the user can naturally rotate their head to scan for signs, and the enhanced information may be integrated directly with their remaining vision. Existing HMD devices for people with low vision can perform overall contrast enhancement and magnification via real-time near-eye video displays [18,19]. Other proposed systems use visual multiplexing to expand the visible visual field [20]. However, commercially available HMD systems for low vision remain expensive and none are currently in wide adoption.

Moving forward, tools that allow for visual enhancement via off-the-shelf hardware hold promise to increase the utility of HMDs for users with reduced vision. In particular, augmented reality (AR) via optical see-through HMDs may allow for task-specific information to be provided in a way that does not completely occlude the user’s natural vision [21–23]. Recent studies have confirmed the visibility of text and patterns on see-through displays even when vision is reduced [21,23]. At the same time, other recent work has explored creating customized AR cues to assist users with a specific task (product search), and found that these cues allowed participants with low vision to substantially reduce their search times [24]. The possibilities, pitfalls, and best practices for AR-based visual enhancement across different tasks is an important area of inquiry for broadening the utility of AR as an assistive tool.

Here, we describe the design and implementation of an application for a consumer AR platform (Microsoft HoloLens) that provides indoor wayfinding assistance directly in the user’s natural field of view. The system leverages recent advances in computer vision and HMDs to allow visually impaired users to interact with existing wayfinding infrastructure (text-based signs), without modification to the physical environment. Specifically, the system uses optical character recognition (OCR) to identify signs, which are then localized in 3D and visually enhanced in AR. In a behavioral experiment, participants with simulated visual impairment were asked to use the system while locating a particular office within a hallway. The results suggest improvements in subjective task assessment and route efficiency – but overall negative effects on task speed – as compared to a control group. The conclusions drawn from the current study can be used to inform future work on AR visual assistance.

## Materials and methods

### System design and implementation

**Fig 1:**
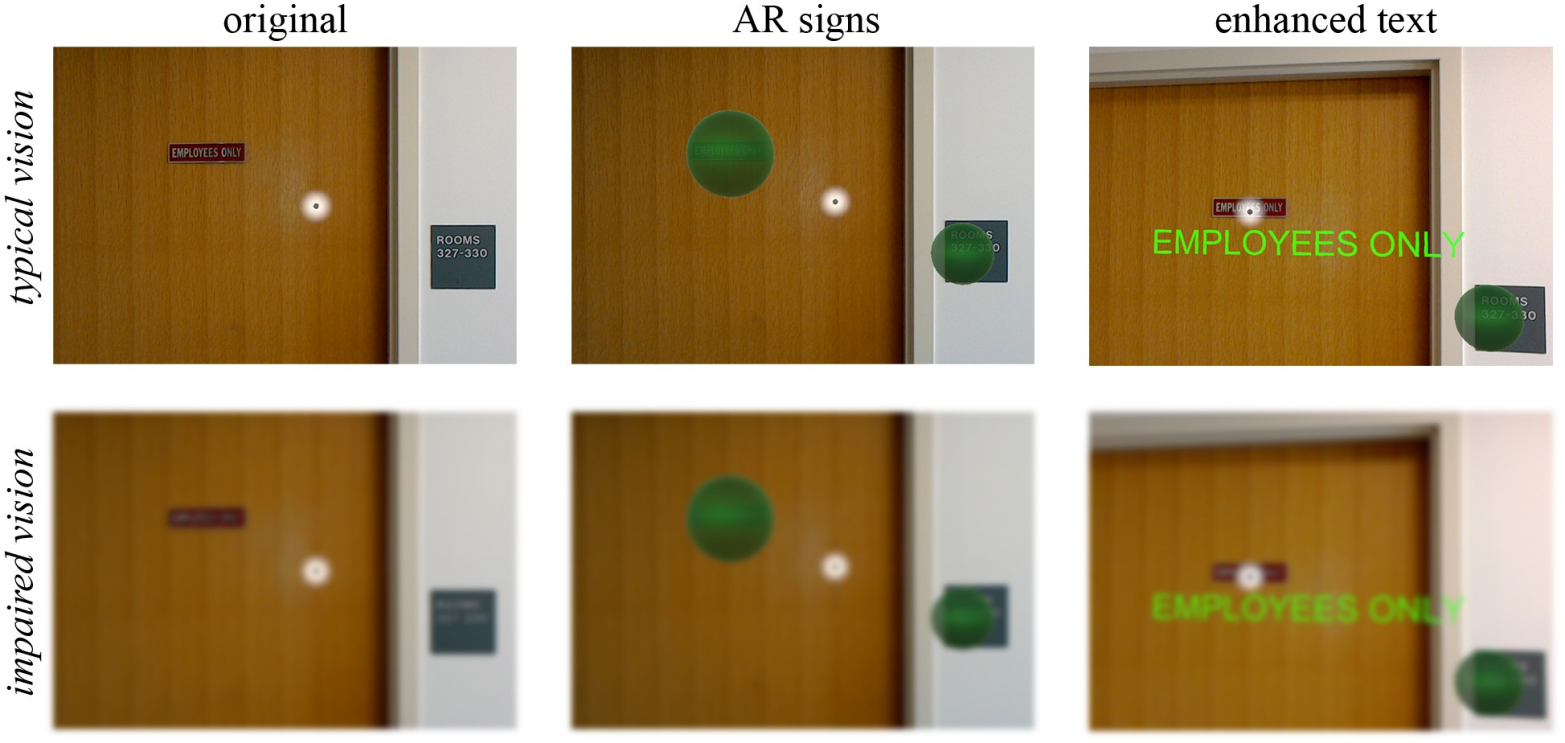
Application screenshots. Left column: Original view of a user looking at two signs with normal vision (top row) and simulated visual impairment via Gaussian blur (bottom row). A white circle indicates the user’s virtual cursor. Note that the signs are no longer readable with visual impairment. Middle column: Green AR signs are shown placed over the physical signs, indicating that the application identified two signs with high certainty. In real-time, these signs would be flashing at 3 Hz. Right column: Text is displayed with enhanced visibility after a user selects an AR sign.

Fig 1 provides an overview of the application, showing the user’s point of view through the optical see-through HMD (see Hardware). Panels illustrate the appearance both with normal vision (upper row) and a simulated visual impairment (lower row). The white dot indicates the location of a virtual “cursor” that follows the user’s head movement and can be used to select a visual region to be searched for signs (left column). When signs are detected in the environment (via optical character recognition; OCR), flashing AR indicators appear (middle column). The indicators are anchored to the 3D location of the sign and remain fixed when the user moves around. Thus, the indicators allow the user to visually identify signs by making them highly salient. The user can select individual AR signs and the application will display the text content in magnified, high-contrast font (right column) as well as read it aloud via text-to-speech.

### Hardware

Microsoft HoloLens is an untethered optical see-through HMD system designed to support AR for consumer and enterprise applications [25]. The sensors include a 2.4 megapixel camera, inertial measurement unit, and infrared depth camera, which allow the HoloLens to track the user’s movements and map the 3D geometry of the environment. For user interaction, the system has gesture recognition, voice control, Wi-Fi and Bluetooth connectivity, as well as speakers. A virtual cursor enables users to select particular spatial locations – the cursor tracks the location directly in front of the user’s head. In the current study, we used a small Bluetooth clicker that accompanies the development edition.

### Software

Software development was performed using Microsoft’s HoloToolkit and Unity (Unity Technologies). When looking for a sign, the user initiates OCR either by pressing the clicker or by saying “What’s here?”. On this command, the PhotoCapture API in Unity captures an image with the HoloLens’ front-facing camera (45° horizontal field of view, 1280 × 720 pixels, 32bit RGB format). The application constructs a JavaScript Object Notation (JSON) request containing this image and sends it to the Google Cloud Vision API, a cloud-based computer vision engine, over Wi-Fi. The response from the API contains a series of entries, each with a description and pixel bounding box for a region of text detected in the image. This process takes 3–4 sec on average.

If the response is empty, the user is notified with synthetic speech: “No text was detected.” If the response is not empty, there is no guarantee that separate response entries from the API correspond to physically separate signs. For example, a sign for Room 323 might in some instances return two entries (“Room” and “323”), which should ideally not result in two separate AR signs. Thus, if the distance between multiple response entries within a given image capture falls below a specified threshold (in pixels), the text is combined and allocated to the same AR sign. In the current implementation, this threshold is set to 30 pixels.

To determine the 3D location of each sign in physical space, a virtual camera is placed at the location and orientation that the picture was captured from, using the on-board motion tracking. A ray is cast from this camera through the center of the pixel bounding box, and the intersection with the 3D map of the surroundings is recorded. Note that this 3D map is constructed in real-time via the depth sensor, not loaded from an external source. Importantly, each time a new image is captured, any new AR signs must be compared to the existing signs to avoid generation of duplicates. Signs are likely to be duplicates when a new sign has both similar text and similar 3D coordinates to an existing sign. To prevent duplicates, locality hashing is performed on all sign texts, and the application discards any new signs with similar text and similar 3D coordinates to an existing sign. In the current implementation, a distance threshold of 13.5 cm is used, such that signs with similar text that are less than 13.5 cm from an existing sign are considered duplicates. This threshold was determined during pilot testing. This method accounts for minor errors in the OCR, as well as offsets that can occur due to errors in motion tracking as the user moves around an environment.

The next step is to create highly visible AR sign indicators. This is done by placing a virtual sphere at the location of the text. As the user continues to move, the world coordinates of the sphere stay the same, creating an AR sign in the same location as the real sign. The diameter of the sphere matches the maximum dimension of the text in the world, and each sphere flashes at 3 Hz to improve visibility. Indicators are also colored based on the confidence that the associated text has been accurately recognized, using the height of the bounding box in pixels. For the current implementation, signs with text height >20 pixels, 11–20 pixels, and ≤ 10 pixels were colored green, orange, and red, respectively. When the user sets the cursor on a sign, the indicator stops flashing and turns bright magenta. Pilot tests confirmed that the spheres, colors, and cursor were all visible with visual acuity of ~1.30 logMAR (~20/400), although color vision deficits could impede users from distinguishing between confidence colors.

The user has the option to either read all AR signs at once or select a single sign. To read all of the AR signs, the user says “Read all here,” which reveals the enhanced text of every sign in the current field of view. Alternatively, the user can select an individual sign with the cursor, and say “Show me” or press the clicker. When a sign is being read, the sphere is hidden and replaced by bright green Arial font subtending 2° vertically (Fig 1 right column). By default, text-to-speech is also used to read the text aloud. To dismiss the current sign, the user can either say “Hide words” or press the clicker again; the AR sign then reappears. Source code for the application is publicly available online at http://github.com/eacooper/HoloLensSignARApp.

### Study participants

Twenty-four undergraduate students with normal or corrected-to-normal vision took part in the user study (15 female, mean age 19.5 yrs). All participants wore swim goggles modified with Bangerter occlusion foils (two layers of LT 0.1 foils) that reduced their vision to 1.28 logMAR on average [26]. Participants were randomly assigned into two groups, and completed a short navigation task (finding a specific office in a hallway with several doors). The *control group* completed the task without any assistance, and the *AR group* used the sign-reading application. Both groups wore the HoloLens while completing the task, because the built-in motion tracking was used to record walking trajectories. Informal testing suggested that the HoloLens visor slightly, but not substantially, reduced visual acuity as measured in typical indoor lighting. All participants gave informed consent, and the protocol was approved by the institutional review board at Dartmouth College.

### Study procedure

Participants started at one end of an unfamiliar hallway (2.1 m × 27 m) that contained 12 doors, including 10 offices with name and room number signs, and two restrooms also with signs. They were asked to find a particular professor’s office in the hallway, located approximately 16.7 m from the starting position. They were given the professor’s name, and were unaware of the office location prior to performing the task. The AR group practiced using the system briefly in a separate room before performing the task. After completing the task, participants were asked to consider times when they have had to locate a room in an unknown building, and rate their agreement with three statements from 1(low) to 5(high) based on their experience in the study: “This task would be easy,” “I would feel comfortable doing this task in a typical indoor environment,” and “I would feel confident in my ability to perform the task.”

The HoloLens motion tracking recorded the participant’s position during the task at approximately 50 Hz. To determine average walking speed, for each participant we first calculated the distance travelled between each pair of consecutive position measurements. Data points were excluded in which participants were stationary (speed < 0.005 m/s). We also excluded data points with high speeds that were likely due to recording artifacts (speed > 5 m/s). The average speed was then computed for each participant from the remaining measurements.

While our primary focus was to compare the control and AR groups, we also wanted to examine whether individuals preferred doing this task with or without the application. Thus, after giving their responses, the control group repeated the task using the sign-reading assistant, and the AR group repeated the task without it. The second trial was conducted in a different hallway. Afterwards, participants reported which trial was easier, and on which trial they were more comfortable and confident. Raw motion tracking and response data for each participant, as well as plotting and analysis code, are included in S1 Appendix.

## Results

**Fig 2:**
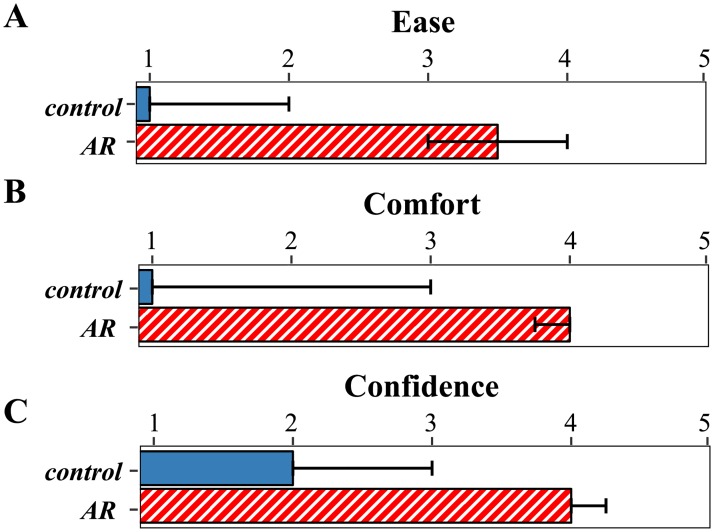
Subjective responses. Bars show median and interquartile range for ratings of ease (A), comfort (B) and confidence (C) in the two groups.

All participants completed the task successfully. The agreement ratings for the three statements were assessed using a Wilcoxon rank sum test, in order to determine whether the distribution of ratings differed between the control and AR groups. According to this analysis, the AR group reported that they found the task easier (*W* = 151, p < 0.001), and felt more comfortable (*W* = 138, p < 0.001), and confident (*W* = 141, p < 0.001) than the control group (Fig 2). After both groups completed the task a second time for comparison, 96% of participants preferred the AR trial for ease, 83% for comfort, and 92% for confidence. While these results all reflect subjective responses, they suggest that the AR signs may provide a usable and potentially helpful tool for navigating to an unfamiliar location via text-based signage.

**Fig 3:**
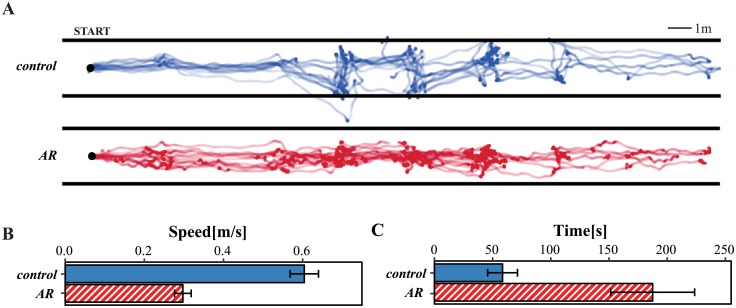
Path analysis. (A) Lines show estimated paths taken by users in the control (blue) and AR (red) groups. The width of the hallway is illustrated for each group with two horizontal lines. Note that paths outside of the hallway suggest errors either in motion tracking or path alignment. (B, C) Bars show mean and standard error for walking speed and time to complete task. Note that walking speed excludes stationary periods.

We also examined how the two groups moved through the hallway and how often they initiated the OCR. Fig 3A shows all individual walking trajectories of the control (blue) and AR (red) groups (paths were registered across trials by aligning the axis of maximal variance). Overall, the control group had to get closer to signs in order to read them and therefore often “zig-zagged” along the hallway, whereas the AR group moved on more efficient trajectories down the center of the hall. In an exploratory analysis, we quantified these differences in trajectory with a distance ratio for each participant: the distance travelled across with width of the hallway relative to the distance travelled along the length of the hallway. Smaller ratios suggest a more direct trajectory along the length of the hallway (however, not necessarily always in the forward direction), and larger ratios suggest more time spent zig-zagging. This analysis supported the observation that the AR group (mean = 0.36, standard deviation = 0.09) travelled back and forth across the width of the hallway relatively less than the control group (mean = 0.50, standard deviation = 0.10). The difference in group means was statistically significant, as assessed with a t-test (t(21.84) = -3.67, p < 0.001).

Despite the more direct paths taken by the AR group, this group on average walked at a slower speed and needed substantially more time to complete the task, relative to the controls (Fig 3B and 3C). The differences in group means for walking speed and task completion time were statistically significant, as assessed with t-tests: speed ((t(17.75) = -7.39, p < 0.001)) and time (t(13.34) = 3.37, p < 0.005). Note that degrees of freedom here reflect the use of Welch’s t-test, which was employed because the variances were not equal between groups for both walking speed and time to complete the task. The finding of longer walking time when using the AR application is consistent with prior work, which showed that people with typical and low vision walked slower while performing a task using AR glasses [21].

In this analysis, task duration takes into account the amount of time that the participants were walking, and the amount of time that they were stationary. Thus, the increased task duration can likely be attributed to two factors: the overall slower walking speeds relative to the control group and the processing time needed to perform OCR (leading to more stationary time). On average, participants in the AR group initiated the OCR 9.8 times during the task, with a minimum of 3 and a maximum of 22. Based on the average processing time (see Methods), this suggests that participants spent an average of 30–40 s waiting for the OCR result to return. While this is still less than the difference in completion time relative to the control group (which was 129 s), it suggests that faster OCR has the potential to substantially increase user efficiency. However, shorter response times may also alter the way that users interact with the application (e.g., increase the number of signs that they read).

## Discussion

The results of the behavioral study suggest that an AR-based sign-reading assistant may provide a useful tool for increasing ease, comfort and confidence when visual wayfinding is impaired. However, there are several limitations in the current system that can be addressed with future work.

First, the application relies on a cloud-based service for OCR; as such, it requires a wireless internet connection and takes several seconds to obtain results. Second, while the 3D localization of the signs was relatively accurate, there were clear cases in which the AR sign did not overlap with the physical sign. While these errors were not substantial enough to reduce the users’ ratings of the task relative to the control group, localization could be improved with higher resolution (spatial and temporal) 3D mapping and/or motion tracking. In addition, the current system does not have an efficient solution for areas with a lot of signage, which could lead to an obtrusive number of blinking indicators, or enhanced text that is too long to comfortably read on the screen. Third, the current application only searches for signs when the user manually initiates it, and as such requires visual guidance [27]. Future work can explore integrating text recognition approaches that run locally and still have high accuracy – and examine whether it is desirable to automatically and continuously search for signage in the environment as opposed to using manual interaction. Finally, participants in our study wore low vision simulators and, thus, never experienced visual impairment outside of the experiment. Users who are visually impaired in day-to-day life may respond differently to the application. However, low vision simulators are often employed to examine task performance in controlled settings [23,28,29]. In the current study, the use of simulators allowed us to perform between-subject comparisons with control and AR groups that had essentially identical levels of visual ability and experience with the task. Examining how differing levels of low vision, experience, and other user demographics impact usability will be important moving forward, as well as leveraging the ability to customize visual appearance in AR to suit different users.

In conclusion, consumer AR systems hold the potential to augment visual information for everyone. In the process of developing these technologies, it is essential to consider the needs of and opportunities presented for users with a range of different levels of vision. While consumer AR systems are not yet common place in day-to-day life, our work suggests that AR provides a promising and flexible future platform to address gaps in common assistive tools.

## Supporting information

S1 Appendix Raw data and example code. (ZIP)
