# Supplementary figures and images for: An augmented reality sign-reading assistant for users with reduced vision
Source: PLoS One. 2019 Jan 16;14(1):e0210630. doi: 10.1371/journal.pone.0210630 (PMC6334915; doi:10.1371/journal.pone.0210630)

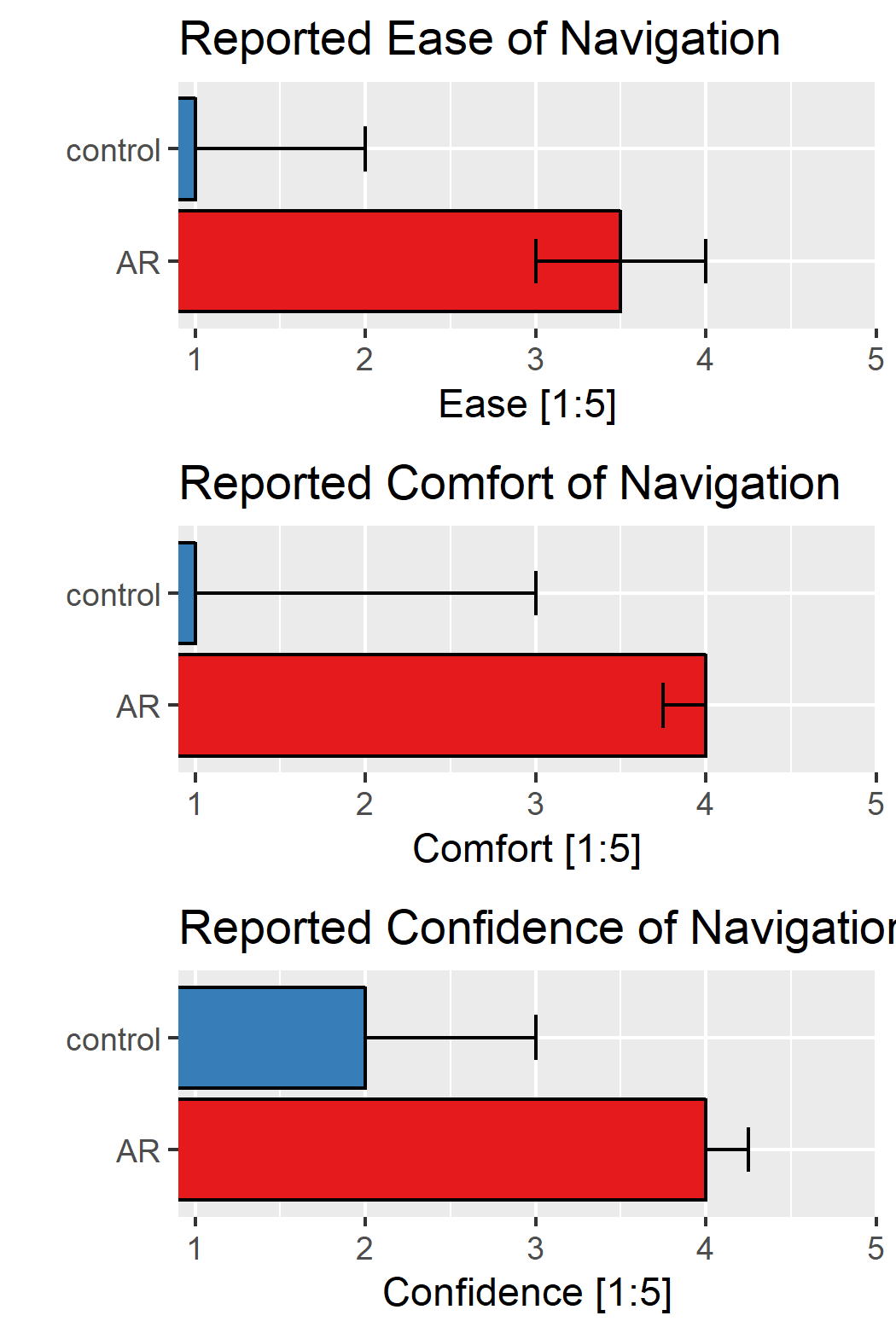

Supplement: S1 Appendix — (ZIP) [file pone.0210630.s001.zip › S1 Appendix/Plots/ratings.png]

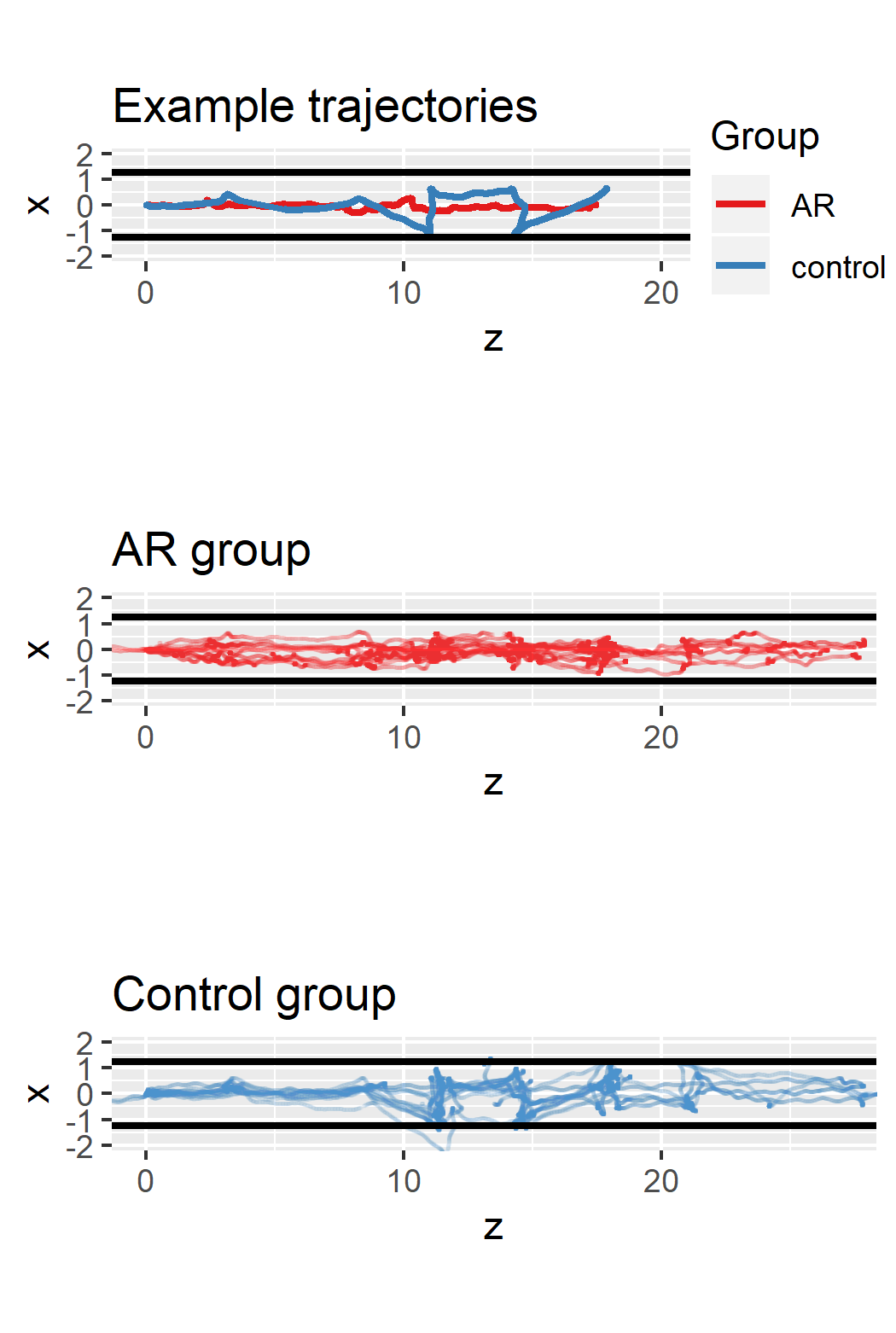

Supplement: S1 Appendix — (ZIP) [file pone.0210630.s001.zip › S1 Appendix/Plots/trajectories.png]
